# Special Issue on Biotechnological Applications of Oxidoreductases

**DOI:** 10.3390/ijms25031758

**Published:** 2024-02-01

**Authors:** Maria Camilla Baratto, Rebecca Pogni

**Affiliations:** Department of Biotechnology, Chemistry and Pharmacy, University of Siena, 53100 Siena, Italy; mariacamilla.baratto@unisi.it

This Special Issue was launched in conjunction with the 10th edition of the OxiZymes meeting in Siena (Italy) in 2022. Initially dedicated specifically to research on laccases and peroxidases, this series of conferences now cover the entire class of oxidoreductases. Oxidoreductases, which are enzymes catalyzing redox reactions, comprise a large number of enzymes of industrial relevance, including peroxidases, peroxygenases, laccases, flavin-containing oxidases, dehydrogenases, unspecific peroxygenases (UPOs), dye-decolorizing peroxidases (DyPs), and copper-containing lytic polysaccharide monooxygenases (LPMOs). Research on oxidoreductases covers different aspects ranging from discovering novel enzymes and conducting structure–activity relationship studies to exploring their applications in the production of fine chemicals and polymer building blocks, biosensors, and biomaterials for establishing a bio-based economy [1]. In chemical synthesis, oxidoreductases play an important role; the reaction routes are shorter, and for many of them, the chemical counterpart does not exist [2]. Furthermore, the generally mild reaction conditions may also reduce the environmental impact of the biocatalytic reactions compared to classical counterparts.

To cite some examples, laccases are copper-containing metalloenzymes that can be employed for the synthesis of fine chemicals, textile dyes, and development of novel green organic transformations [3,4,5]. Laccases engineered by directed evolution can also be applied for Kraft pulp biorefineries and fiberboard manufacture [6,7] whereas the mono-copper enzymes, currently named Lytic Polysaccharide MonoOxygenases (LPMOs), have intriguing and unprecedented catalytic properties. They can act as monooxygenases and peroxygenases in the oxidative cleavage of recalcitrant polysaccharides like cellulose and chitin [8]. Simultaneously, engineered flavoprotein oxidases exhibit interesting properties suitable for their use in industrial applications, as well as Unspecific Peroxygenases (UPO) and dye peroxidases (DyPs) [9,10,11].

In this Special Issue, papers related to the different biotechnological applications of oxidoreductases are reported.
